# Participation of health volunteers in identifying, reporting and solving health and safety problems in the Sahand city in the year 2017-2018 (a community-based experience) 

**Published:** 2019-08

**Authors:** Roghayeh Javadi Savojbolaghi, Raheleh Soltani, Tofigh Alizadeh Mobasher, Oldoz Tarvirdi, Raheleh Ghasemzadeh, Roghayeh Berejfrosh Azar, Khadijeh Rezaei

**Affiliations:** ^*a*^East Azarbaijan Health Center, Health Education and Health Promotion Expert, East Azarbaijan, Iran.; ^*b*^Departman of Health Education and Health Promotion, Arak University, Arak, Iran.; ^*c*^East Azarbaijan Health Center, Head of Education and Health Promotion Department, East Azarbaijan., Iran.; ^*d*^Tabriz Health Center, Health Education Expert, Tabriz, Iran.; ^*e*^Osko Health Center, Health Education Expert, Tabriz, Iran.; ^*f*^East Azarbaijan Health Center, Head of Department of Education and Health Promotion, East Azarbaijan, Iran.

## Abstract

**Background:**

Based on the recognition that the actual participation of society is essential for the development of that society, the concept of social mobilization originates. Social mobilization is in fact a tool that enables people to organize themselves through collective action in order to achieve their goals. Without the participation of people, reaching the goals of health and solving the safety problems is unexpected. Organizational forces, including health volunteers, are important social resources that health experts around the world emphasize on their planned participation in community health promotion programs. In Iran, the use of women as health volunteers has begun to meet the health promotion needs of the 1990s. In this regard, health volunteers who have taken steps to promote and develop community health have been used in the new city of Sahand to reduce the risk of traffic jams.

**Methods:**

The program included a social co-operation / social co-operation model as well as a guide to empowering citizens and neighborhoods to promote health. The program was conducted in Sahand City in 2017-2018 with the participation of 410 health volunteers.

**Results:**

After the necessary coordination meetings and trusteeship meetings were held, a list of problems and the establishment of external meetings were taken to correct the traffic safety problems by the relevant departments.

**Conclusions:**

It is possible to sensitize, justify and raise awareness among health volunteers as well as empowerment through community-based initiatives through education and capacity building with community health resources.

**Keywords:**

Health volunteers, Partnerships, Community-based initiatives

